# Supplementary material for: Distinct involvements of the subthalamic nucleus subpopulations in reward-biased decision-making in monkeys
Source: eLife. 2026 Jul 1;15:RP109622. doi: 10.7554/eLife.109622 (PMC13322701; doi:10.7554/eLife.109622)
Supplement: Peer review history

# Editor report: eLife Assessment

**DOI:** 10.7554/eLife.109622.4.sa0

**Authors:** Timothy D Hanks

**Affiliations:** University of California, Davis United States

**Keywords:** Convincing, Important

This study presents analyses of single neuron activity in the subthalamic nucleus (STN) of monkeys performing a decision-making task that manipulates both perceptual evidence and reward. The study shows **convincing** evidence of distinct subpopulations of neurons in STN that differ in their representations of key quantities related to decision formation. These findings reveal **important** functional heterogeneity within the STN that helps provide new insights into its contributions to decision processing.

# Referee report: Reviewer #1 (Public review)

**DOI:** 10.7554/eLife.109622.4.sa1

[Editors' note: this version has been assessed by the Reviewing Editor without further input from the original reviewers. The authors have addressed the comments raised in the previous round of review.]

Summary:

This manuscript offers a careful and technically impressive dissection of how subpopulations within the subthalamic nucleus (STN) support reward-biased perceptual decision-making. The authors recorded STN neurons in monkeys performing an asymmetric-reward visual motion discrimination task, then combined single-unit analyses, regression modeling, and drift-diffusion model (DDM) fitting to identify functionally distinct neuronal clusters. Each subpopulation shows unique relationships to computational decision variables - evidence accumulation rate, decision bound, and non-decision time - as well as to post-decision evaluative signals including choice accuracy and reward expectation. The revised manuscript substantially strengthens the original submission by improving both the objectivity of neuron selection and the robustness of the clustering solution.

Strengths:

The asymmetric-reward paradigm cleanly separates perceptual and motivational contributions to STN activity, allowing the authors to characterize how neurons blend these distinct sources of information. The dataset is extensive and well-controlled, and the behavioral and neural analyses are tightly integrated. Relating cluster-specific activity to DDM parameters provides an interpretable computational link between population signals and behavior. The clustering solution is now validated across two algorithms, two monkeys, and subsets of trials - establishing that the three-cluster structure is robust. The new Figure 9 offers a conceptually useful, if necessarily speculative, synthesis connecting the identified subpopulations to distinct basal-ganglia pathways (hyperdirect versus indirect). The new Figure 8 documenting the anatomical intermingling of subpopulations is also important, as it directly informs the interpretation of prior and future STN stimulation studies.

Weaknesses:

The inferred relationships between neural clusters and DDM parameters remain correlational - the authors now appropriately flag this throughout, and the causal inference gap is acknowledged in the Discussion with concrete proposals for future targeted perturbation strategies. While a generative multi-cluster model would further strengthen mechanistic interpretation, the conceptual framework in Figure 9 provides a reasonable intermediate step given the scope of the study and the absence of simultaneous population recordings, which preclude direct inter-cluster covariation analyses. These remaining limitations are inherent to the experimental design rather than analytical oversights.

Comments on the previous version:

The authors have responded thoroughly and constructively to all of my concerns. The revised clustering pipeline - incorporating finer temporal resolution, objective neuron selection, outlier removal, a second clustering algorithm, cross-monkey validation (Rand indices of 0.94 and 1.0 for the two monkeys), and trial-subset stability analysis - substantially increases confidence in the three-cluster solution. The correlational nature of the DDM-activity relationships is now clearly stated, and the Discussion appropriately contextualizes the causal inference gap while suggesting feasible future directions. The new Figure 9 provides the conceptual synthesis I had hoped for, within the realistic scope of the present study. I am satisfied with the authors' responses and have no further requests.

# Referee report: Reviewer #2 (Public review)

**DOI:** 10.7554/eLife.109622.4.sa2

This study uses monkey single-unit recordings to examine the role of the STN in combining noisy sensory information with reward bias during decision-making between saccade directions. Using multiple linear regressions and clustering approaches, the authors overall show that a highly heterogeneous activity in the STN reflects almost all aspects of the task, including choice direction, stimulus coherence, reward context and expectation, choice evaluation, and their interactions. The authors report in particular how three classes of neurons map to different decision processes evaluated via the fitting of a drift-diffusion model. Overall, the study provides evidence for functionally diverse and anatomically intermingled populations of STN neurons, supporting multiple roles in perceptual and reward-based decision-making.

This study follows up on work conducted in previous years by the same team and complements it. Extracellular recordings in monkeys trained to perform a complex decision-making task remain a remarkable achievement, particularly in brain structures that are difficult to target, such as the sub-thalamic nucleus. The authors conducted numerous analyses of STN activities, using sophisticated statistical approaches and functional computational modeling.

# Author comment: Author response

**DOI:** 10.7554/eLife.109622.4.sa3

**Authors:** Kathryn Branam, Joshua I Gold, Long Ding

**Affiliations:** University of Pennsylvania Philadelphia United States; University of Pennsylvania Philadelphia United States; University of Pennsylvania Philadelphia United States

The following is the authors’ response to the previous reviews

**Public Reviews:**

**Reviewer #2 (Public review):**

One criticism that I would still make in the revised version of the paper concerns the description of the behavior of the two monkeys which is still minimal, while acknowledging differences in their choice and RT performance that reflect "individual differences in sensitivity to motion stimulus and a common heuristic-based satisficing strategy". This sentence is not clear to me. Moreover, the potential consequences of these differences on neuronal activity are only considered in the cluster analysis done for each of the two animals separately and for which it turns out there is no notable difference.

We have revised the text to emphasize the key, common feature of their behavior and refer readers interested in variability across sessions and individuals to our previous study: “Both monkeys showed consistent biases toward the large-reward choice (Figure 1B, C). Details of their performance, including variations across sessions and individuals, have been reported in a previous study (Fan et al., 2018).”

Given that both monkeys’ choices and RT showed clear and consistent coherence and reward dependencies, and that the clustering analysis were consistent across the two monkeys, we believe that our analyses presented here are appropriate. Future work is needed to examine if and how STN contributes to more nuanced aspects of behavioral variability.

Compared to the first version of the paper, the cluster analysis in this revised version yields three distinct populations instead of the previous four. While the authors suggest that these subpopulations play important roles in encoding different aspects of decision-making, the identification of three rather than four subpopulations seems to me an important update that warrants discussion.

The clustering results are slightly different because, following suggestions from the first round of reviews, we now use more principled approaches for selecting neurons and computing the clusters. The primary difference is that Clusters 1 and 3 in the original manuscript have mostly been merged into one cluster (new Cluster 3). We updated the text to note that our use of three clusters depends on our choice of clustering cutoff and continue to emphasize that the clusters are consistent across monkeys and clustering techniques: In Results: “Inspection of the dendrogram (hierarchical cluster tree) suggested that our STN samples can be reasonably grouped into three clusters, although other groupings are possible using different clustering cutoffs (Figure 5-S1).” In Discussion: “Furthermore, our clustering analysis aimed to identify common activity profiles in the STN population, while leaving behind many neurons that either did not show consistent task-related modulation or had less common activity profiles (e.g., those that were far from others in the vector space and those with too infrequent occurrence to form detectable clusters). More work is needed to continue to refine our understanding of the specific computational contributions of the STN to decision formation.”

Finally, I think it would have been interesting to identify the level of collinearity in the model proposed by the authors (equation 7). Indeed, one can expect significant collinearity between some of the proposed explanatory factors of neuronal activity, such as choice and coherence level, for example.

The reviewer is correct that choice and coherence are correlated with the formulation of Eq. 7. However, such collinearity does not seem to bias the regression results (Author response image 1). We have performed simulations with different modulation strengths and noise levels (A and C) and observed generally good recoverability of the ground-truth regression coefficients (red: unity-slope lines), despite the strong correlation between choice and coherence for one choice (B).

**Author response image 1.**

Similarly, for the analysis relating neuron activity to decision evaluation signals (p 16), firing rates calculated using sliding averages with 1-ms steps are compared, but the method does not specify controls for multiple comparisons or for non-independent data.

We have made multiple comparison corrections using the Benjamini and Hochberg procedure and updated the relevant text in Methods, Results, and Abstract accordingly.
